# Organic Fertilizer Application Mediates Tomato Defense Against *Pseudomonas syringae* pv. *Tomato*, Possibly by Reshaping the Soil Microbiome

**DOI:** 10.3389/fmicb.2022.939911

**Published:** 2022-06-21

**Authors:** Feng Huang, Chunhao Mo, Linfei Li, Jingling Shi, Yiwen Yang, Xindi Liao

**Affiliations:** ^1^College of Animal Science, South China Agricultural University, Guangzhou, China; ^2^Guangdong Provincial Key Lab of Agro-Animal Genomics and Molecular Breeding, Key Laboratory of Chicken Genetics, Breeding and Reproduction, Ministry Agriculture, Guangzhou, China; ^3^National-Local Joint Engineering Research Center for Livestock Breeding, Guangzhou, China

**Keywords:** organic fertilizers, foliar pathogens, microbial diversity, tomato, disease suppression, biological control

## Abstract

Bacterial speck caused by *Pseudomonas syringae* pv. *tomato* is a serious foliar disease on tomato. However, it is still unknown how organic fertilizers application mediates plant defense against foliar pathogens by altering the composition of the soil microbial community. We conducted a 2-cycle pot experiment involving chemical and organic fertilizers and tracked tomato foliar pathogen incidence. Using microbiome sequencing, we then compared the differences in bulk and rhizosphere microbial communities. The results showed that, compared with soils amended with chemical fertilizer, soils amended with organic fertilizer gradually and significantly presented a reduction in tomato foliar disease, and the bacterial richness and diversity significantly increased. Moreover, the bacterial and fungal compositions of the bulk soil and rhizosphere soil of the organic fertilizer and chemical fertilizer treatments were different from each other. More importantly, the abundance of some potentially beneficial bacteria, such as *Luteolibacter*, *Glycomyces*, *Flavobacterium*, and *Flavihumibacter*, increased in the organic fertilizer-amended soil, and these genera were significantly negatively correlated with the incidence of tomato foliar disease. These results suggest that organic fertilizers can alter the taxonomy of the soil microbiome and that some specific beneficial microbial communities may play an important role in reducing the infection of foliar pathogens by inducing plant resistance.

## Introduction

Large amounts of chemical fertilizers (CFs) are applied to soils, causing various major threats, such as soil acidification, nitrogen leaching, a decrease in soil organic matter and an increase in plant diseases (Bailey and Lazarovits, 2003; Xu et al., 2020). Consequently, the effect of CFs on increasing crop yields diminishes over time (Horrigan et al., 2002). In addition, chemical pesticides used to protect crops from insects and pathogenic bacteria can cause environmental pollution (Rajmohan et al., 2020). In this context, organic fertilizers (OFs) that have the potential to improve soil properties, improve nutrient use efficiency and maintain plant health are being reconsidered as plant fertilizers (Liu et al., 2017; Yang et al., 2019). OFs are kinds of fertilizer made of byproducts rich in organic matter, such as animal manure or plant residues, as the main raw materials. Organic waste materials (e.g., animal manure) can be made into OFs that not only solve the problem of environmental pollution but also act as soil amendments (Lutz et al., 2020). Many studies have shown that the application of OF can increase the soil organic matter content and alter the structure and function of microbial communities (Sun et al., 2015; Tao et al., 2017; Lin et al., 2019). More importantly, OF improves the ability of plants to resist infection by pathogenic bacteria.

In agricultural production, one method to overcome the occurrence of pests and diseases involves the improvement of soil properties by fumigation, crop rotation and OF application (Larkin, 2008; Bonanomi et al., 2010; Ge et al., 2021). Soil fumigants can kill pathogenic bacteria and cannot improve the physical or chemical properties of the soil; moreover, these fumigants can also kill beneficial microorganisms at the same time. Agronomic planting methods such as crop rotation, despite being simple and environmentally friendly, often take several years to exert a certain effect, and sometimes the effect is not substantial. The application of OFs can activate soil nutrients and increase soil nutrient contents. More importantly, OFs can effectively control most soil-borne pathogens (Bailey and Lazarovits, 2003). The OFs capacity to suppress plant diseases is primarily associated with the biological activity and biomass of its microbiota (De Corato, 2020). Bonilla reported that the application of sterilized OF resulted in a decreased ability to suppress disease (Bonilla et al., 2012). A recent study showed that organic management can inhibit aboveground insect pests indirectly by altering soil microbial communities, a phenomenon known as induced systemic resistance (ISR; Blundell et al., 2020). Moreover, Berendsen et al. (2018) demonstrated that *Arabidopsis thaliana* specifically promotes *Microbacterium*, *Stenotrophomonas* and *Xanthomonas* spp. in the rhizosphere upon foliar defense activation by downy mildew-causing pathogens. Bacterial speck disease by *Pseudomonas syringae* pv. *tomato* is common disease throughout tomato growing areas. It has been found that *P. syringae* pv. *tomato* mainly infects plant leaves, causing plant tissue necrosis, and resulting in 75% of tomato yield loss. The influence of rhizosphere microorganisms on plant diseases has always been a popular but difficult topic in the field of plant pathology research both in China and in other countries. Nonetheless, how OF alters rhizosphere microbiota protect against aboveground plant pathogen attack remains unclear.

In this study, the effect of OF on tomato foliar disease was examined. Experiments were divided into *CF* and OF treatments, and each treatment involved inoculation with *P. syringae* pv. *tomato* DC3000 (*Pst*). The experiment was carried out for 2 cycles, the disease incidence of each cycle was measured, and soil samples were collected to analyze bacterial and fungal community composition *via* high-throughput sequencing at the end of the second cycle. Reviewing both the relationship between OF application and the soil microbiome and the impact on foliar disease is important with respect to applying OFs and foliar disease prevention and control.

## Materials and Methods

### Soil Preparation

The soil used in the study was collected from a field with no history of tomato cultivation in Guangzhou, Guangdong Province, China (23°13′N, 113°81′E), in March 2021. Topsoil samples (depth of 20 cm) were collected, thoroughly mixed and transported in plastic bags to South China Agricultural University, Guangzhou city, Guangdong Province, China. All the soil samples were dried at room temperature and passed through a 3-mm sieve to remove other plant roots and gravel. To restore the microbial community, tomato seedlings were transplanted and allowed to grow for 4 weeks in collected soil, after which the soils were sieved again to remove the plant material. The soil was stored at 4°C until the start of the experiment.

### Experimental Design

A pot experiment was carried out in a climate chamber (25 ± 2°C, 16 h light/8 h dark, light intensity 100 μmol m^−2^ s^−1^, 70% humidity). Two different fertilizer treatments were applied—OF and *CF.* The pot experiment was performed in accordance with a randomized complete block design with 3 replicates for each treatment, each replicate involved 4 pots, and each pot (15 cm diameter and 13 cm height) was filled with 2 kg of soil. OF (chicken manure and mushroom dreg compost) was supplied by LeFu Co., Ltd., Huizhou city, Guangdong Province, China. The test treatment was amended with 40 g of OF and the control treatment was amended with 0.51 g N (urea), 0.44 g P (superphosphate), 0.40 g K (potassium chloride) before each cycle.

Tomato seeds were surface-sterilized with 3% sodium hypochlorite for 3 min followed by 70% ethanol for 1 min and then germinated on water–agar plates for 2 d. The seeds were sown onto seedling plates (each well contained 60 g of seedling substrate). At the three-leaf stage, three tomato plants were transplanted into each pot. At 10 d after transplantation, all the leaves of tomato plants were punctured, and 2 μl of *Pst* suspension (10^7^ CFU mL^−1^; the *Pst* was grown in nutrient broth at 37°C with 220 rpm shaking for 24 h, and the inoculum density was quantified according to colony counts) was inoculated into the wounds. After the *Pst* suspension was allowed to dry naturally on the leaves, pots were randomly placed back into the growth chamber and covered to increase the humidity. Each pot was watered every 2–3 d to maintain the soil moisture content between 60 and 80% of field capacity. Subsequently, all the plants were removed. A 2-cycle tomato plantings were carried out as described above, with the same procedures applied to each cycle. Each successive cycle used soil from the previous cycle after plant removal. Moreover, in the second cycle, the plants received the same concentrations of OF and *CF* as that applied in the first cycle before planting.

### Assay of Tomato Disease Incidence

In each cycle, the disease incidence of tomato leaves was recorded 21 d after inoculation with *Pst*. The typical symptoms caused by *Pst* were the leaves show dark brown specks or black specks that necrotize quickly. Therefore, we defined diseased plants based on the typical bacterial speck disease symptoms observed. The disease incidence was calculated by counting the number of tomato plants with bacterial speck disease symptoms among the total number of plants.

### Soil Sampling and DNA Extraction

Soil sampling was performed at the end of the second cycle. In brief, 5 healthy tomato plants were randomly chosen in each treatment, and the bulk and rhizosphere soil samples were obtained according to the methods of Berendsen et al. (2018). Briefly, tomato roots were gently removed from soil, and the soil adhering to the roots was removed by sterile water (rhizosphere soil), and the bulk soil was collected from the remaining soil. All the soil samples [20 in total: 2 treatments × 5 replicates × 2 compartments (bulk and rhizosphere)] were stored at −80°C until DNA extraction. Briefly, DNA was extracted from the samples using a FastDNA^®^ SPIN Kit for Soil (MP Biomedicals, LLC, Solon, OH, United States) following the manufacturer’s instructions. Subsequently, the concentration and quality of the DNA samples were determined by a spectrophotometer (Qubit^™^ 3, Thermo Fisher Scientific, United States).

### PCR Amplification and Illumina Sequencing

Amplification of bacterial 16S rRNA within the V3-V4 regions was amplified from the soil DNA with the specific primers 341F—806R and a barcode was attached to the forward primer, while the fungal-specific primers ITS1F—ITS2 were used to target the fungal ITS2 region. All the PCR mixtures included 15 μl of Phusion^®^ High-Fidelity PCR Master Mix (New England Biolabs), each primer at 0.2 μm and 10 ng of target DNA. The cycling conditions consisted of an initial denaturation step at 98°C for 1 min, followed by 30 cycles of 98 (10), 50 (30), and 72°C (30 s) and a final 5 min extension at 72°C. The PCR products were mixed in equal proportions, and then, a Qiagen Gel Extraction Kit (Qiagen, Germany) was used to purify the mixed PCR products. The amplicons were sequenced using an Illumina NovaSeq PE 250 platform at Novogene Co., Ltd., Beijing, China.

### Bioinformatics Analysis

Paired-end reads were assigned to samples based on their unique barcodes and were truncated by cutting off the barcodes and primer sequences. Paired-end reads were merged using FLASH (Version 1.2.11),^1^ a very fast and accurate analysis tool designed to merge paired-end reads when at least some of the reads overlap with the reads generated from the opposite end of the same DNA fragment, and the splicing sequences were called Raw Tags. Quality filtering on the raw tags were performed using the fastp (Version 0.20.0) software to obtain high-quality Clean Tags. The high-quality clean tags were compared with a reference database (Silva database^2^; unite database^3^ for ITS) using Vsearch (version 2.15.0) to remove the chimeric sequences. Subsequently, denoising was performed with DADA 2 in QIIME 2 software (version QIIME2-202006) to obtain initial amplicon sequence variants (ASVs), and then, ASVs whose abundance was less than 5 were filtered and removed. The species annotation of all sample readings was performed using QIIME2 software. The absolute abundance of the ASVs was normalized using a standard sequence number corresponding to the sample with the fewest sequences.

The bacterial and fungal diversity were analyzed using the Shannon index, and the Chao 1 index was calculated to evaluate the community richness of the samples. Principal coordinate analysis (PCoA) based on a Bray–Curtis dissimilarity matrix was performed to visualize differences of the samples in complex multidimensional data, and coordinates were used to draw 2D graphical outputs. Analysis of molecular variance (AMOVA) was used to detect the significant differences in microbial communities between the different fertilization management treatments. Afterward, linear discriminant analysis (LDA) of effect size (LEfSe) was performed to determine the significantly abundant bacterial and fungal taxa within the two treatments. Moreover, Spearman rank correlations between the relative abundance of the rhizosphere genera and the disease incidence were used to analyze potentially beneficial microbial genera in the rhizosphere. A co-occurrence network analysis was subsequently performed on the rhizosphere microbiome of each treatment to explore the interactions between ASVs. Additionally, PICRUSt software was used to predict the bacterial metabolic functions by the use of information within the Kyoto Encyclopedia of Genes and Genomes (KEGG) database (Langille et al., 2013).

### Statistical Analyses

Statistical analyses were performed by using IBM SPSS 20.0 software (SPSS, Inc., United States), and statistical tests performed in this study were considered significant at *p* < 0.05. Disease incidence was calculated by the percentage of diseased tomato plants out of the total number of tomato plants in each replicate.

## Results

### Effect of OF on Soil Chemical Properties and Disease Incidence

**Figure 1:**
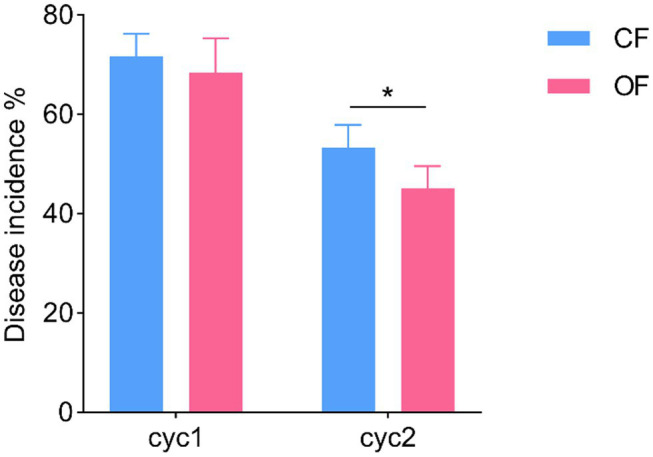
Effects of different fertilization management practices on tomato disease. CF, chemical fertilizer; OF, organic fertilizer. ^*^p < 0.05.

The successive plant experiments showed that OF treatment reduced the incidence of foliar disease in tomato seedlings (Figure 1). In cycle 1, the leaves of tomato seedlings were infected by *Pst*, but the disease incidences were not significantly different (*t* test, *p* > 0.05) between *CF* and OF (Figure 1; Supplementary Table S1). When the cycle 2 tomato seedlings were planted on the previous soil and challenged with *Pst*, the tomato seedlings grown in the OF treatment developed significantly (*t* test, *p* < 0.05) reduced disease symptoms compared to the those of seedlings grown in the *CF* treatment (Figure 1; Supplementary Table S1). In addition, the continuous application of OF also altered the physical and chemical properties of the soil (Table 1). Compared with *CF* treatment, the OF treatment significantly increased the soil electroconductivity (EC), organic matter (OM) content, and available potassium (AK) content. Moreover, Mantel tests showed that soil physiochemical properties were not significantly associated with disease incidence (*r* = 0.12, *p* = 0.07).

**Table 1:** Soil physicochemical properties under different fertilization management programs.

	pH	EC (μS/cm)	OM (g/kg)	AP (mg/kg)	AK (mg/kg)	NH_4_^+^-N (mg/kg)	NO_3_-N (mg/kg)
*CF*	6.92 ± 0.15	121.90 ± 4.34	10.87 ± 0.14	95.52 ± 3.35	404 ± 14.45	8.43 ± 0.91	117.3 ± 9.81
OF	7.19 ± 0.05	302. ± 25.96^***^	13.14 ± 0.5^**^	98. ± 5.24	440.5 ± 8.87^*^	13.76 ± 3.05	120.7 ± 7.41

*CF, chemical fertilizer; OF, organic fertilizer*.

* *p < 0.05;*

** *p < 0.01; and*

*** *p < 0.001*.

### Microbial Diversity and Its Relationship With Disease Incidence

**Figure 2:**
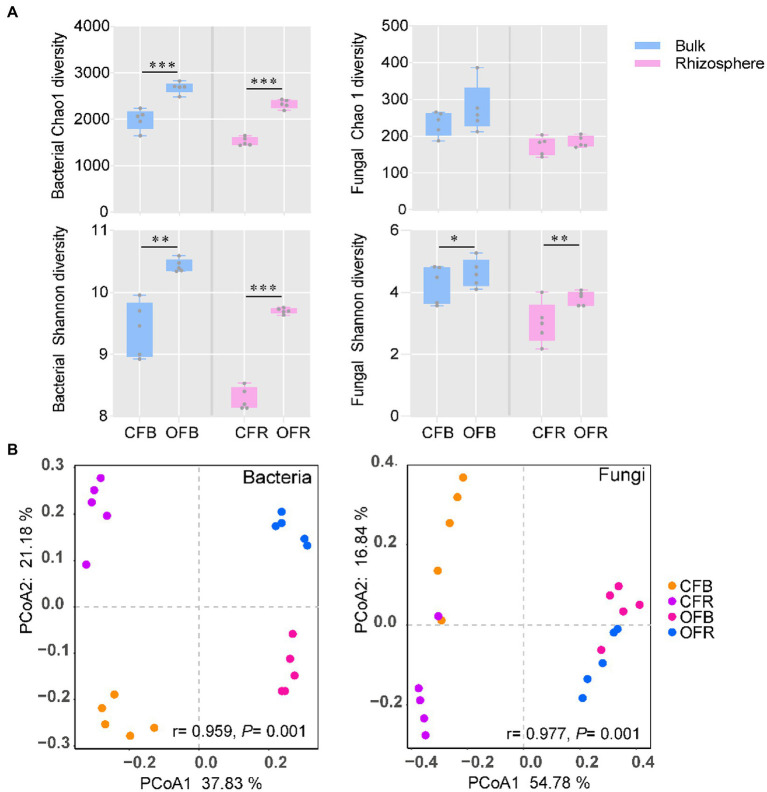
Bacterial and fungal community diversity. **(A)** Boxplot of bacterial and fungal richness and diversity index. **(B)** PCoA ordinations of the bacterial and fungal community compositions based on the Bray–Curtis distance metric for all soil samples. CFB, chemical fertilizer-amended bulk soil; CFR, chemical fertilizer-amended rhizosphere soil; OFB, organic fertilizer-amended bulk soil; and OFR, organic fertilizer-amended rhizosphere soil. ^*^p < 0.05; ^**^p < 0.01; and ^***^p < 0.001.

The diversity of bacteria and fungi under the different fertilization management programs was analyzed *via* 16S rRNA gene and ITS2 region amplification, respectively. In total, 1,688,451 and 1,511,186 high-quality sequences were obtained from bacteria and fungi, respectively. The average read counts per sample containing bacteria and fungi were 75,559 [with a standard deviation (SD) of 5,174] and 84,423 (with a SD of 6,608), respectively. Unlike for fungi, for bacteria, significantly (*t* test, *p* < 0.05) higher bacterial diversity (Shannon) and richness (Chao 1) were observed in organic fertilizer-amended bulk (OFB) soil and rhizosphere soil (OFR). However, the fungal diversity (Shannon) was observed to be higher in the chemical fertilizer-amended bulk (CFB) soil and rhizosphere soil (CFR), but no significant value of fungal richness (Chao 1) was observed between the two different fertilization management programs (Figure 2A). To visualize the differences in microbial communities between different treatments, PCoA based on the detected ASVs was conducted. The PCoA plot showed that there was a significant difference in bacterial (ANOSIM: *r* = 0.959, *p* = 0.001) and fungal (ANOSIM: *r* = 0.977, *p* = 0.001) community compositions under the different fertilization management practices along the first principal component (Figure 2B), the compositions of which were determined to be statistically significant by similarity analysis. Interestingly, the bacterial and fungal communities in the bulk soil were separated (*p* < 0.01) from those in the rhizosphere along the second principal component.

For further analysis, Pearson correlation coefficients between the disease incidence and rhizosphere microbiota showed that the bacterial richness (*r* = −0.70, *p* = 0.03) and diversity (*r* = −0.71, *p* = 0.02) were negatively correlated with disease incidence, while no significant correlations were observed for fungal richness (*r* = −0.49, *p* = 0.15) or diversity (*r* = −0.46, *p* = 0.18; Supplementary Figure S1).

Relationships between microbial taxonomic composition and disease incidence.

Bacterial communities and fungal communities in the bulk and rhizosphere were characterized *via* Illumina NovaSeq sequencing. In general, the majority of ASVs were associated with the phyla Proteobacteria, Actinobacteriota, Bacteroidota, Firmicutes, Acidobacteriota and Verrucomicrobiota across all of the groups, accounting for more than 80% of the total bacterial ASVs (Supplementary Figure S2). In the rhizosphere soil, the phyla Acidobacteriota and Verrucomicrobiota were significantly different in abundance between the OFR and CFR. Moreover, Acidobacteriota and Verrucomicrobiota were also more abundant in the OFB than in the CFB. For the fungi, the ASVs were assigned to five phyla, and only Ascomycota (47.98%) was the predominant fungal phylum across all of the groups (Supplementary Figure S2). Moreover, there was a significantly higher *Ascomycota* abundance in the OFB than in the other treatments.

**Figure 3:**
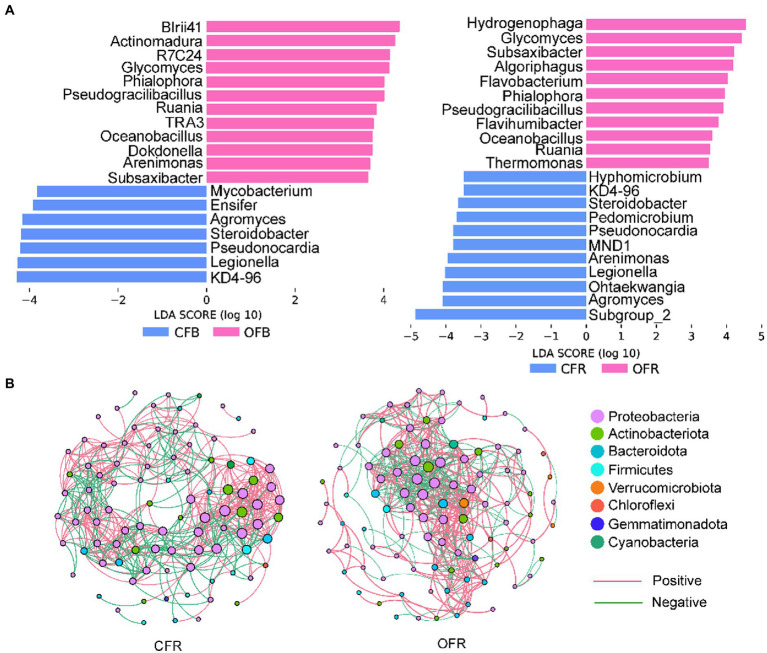
Sensitive genera and co-occurrence network. **(A)** Histogram of the LDA scores calculated for differentially abundant fungus- and bacterium-sensitive genera between the bulk soil and rhizosphere soil. **(B)** Bacterial co-occurrence network of tomato rhizosphere samples.

Considering the importance of the rhizosphere microbial community composition in plant disease resistance, Mantel tests were performed to determine the correlations between bacterial and fungal community composition and disease incidence. The results showed that the rhizosphere bacterial (*r* = 0.274, *p* < 0.05) and fungal (*r* = 0.296, *p* < 0.05) community compositions were significantly correlated with disease incidence. Furthermore, Spearman rank correlation analysis was performed between the relative abundance of sensitive bacterial and fungal genera and disease incidence. A total of 35 sensitive bacterial genera and 1 sensitive fungal genus showed a significant negative correlation with disease incidence in the rhizosphere soil (Supplementary Table S2). The differences in these sensitive genera were analyzed by LEfSe in the bulk soil and rhizosphere soil of different the fertilizer treatments (Figure 3A). Some genera from Bacteroidota and Firmicutes, such as *Subsaxibacter*, *Algoriphagus*, *Flavobacterium*, *Flavihumibacter*, *Pseudogracilibacillus*, and *Oceanobacillus*, were relatively more abundant in the OFR than in the CFR (Figure 3A). In the bulk soil, some genera from the Actinobacteriota, such as *Glycomyces*, *Actinomadura*, and *Ruania,* were relatively more abundant in the OFB than in the CFB (Figure 3A). In addition, *Pseudogracilibacillus* and *Oceanobacillus*, which belong to Firmicutes, were also significantly enriched in the OFB. For fungi, the relative abundance of *Phialophora* in the OFB and OFR was significantly higher than that in the CFB and CFR (Figure 3A). Interestingly, the co-occurrence networks of the rhizosphere bacterial community revealed that *Glycomyces* and *Luteolibacter* were significantly negatively correlated with disease incidence and were found only in the top hub taxa of the OFR (Figure 3B; Supplementary Table S4). Other statistics and the top 10 hub taxa of the CFR and OFR rhizosphere bacterial co-occurrence networks are listed in Supplementary Tables S3, S4, respectively. Mantel tests showed that soil physiochemical properties were significantly associated with bacterial and fungal community composition (*r* = 0.45, *p* < 0.001 for bacteria and *r* = 0.64, *p* < 0.001 for fungi). Furthermore, the relationships between soil physicochemical properties and the sensitive bacteria were analyzed by Spearman rank correlation. As shown in Supplementary Figure S3, the soil pH, AK content, NH_4_^+^-N content, EC, and OM content were significantly positively correlated with the most sensitive microbial genera.

### Different Management Practices Induce Changes in Predicted Rhizosphere Bacterial Functions

**Figure 4:**
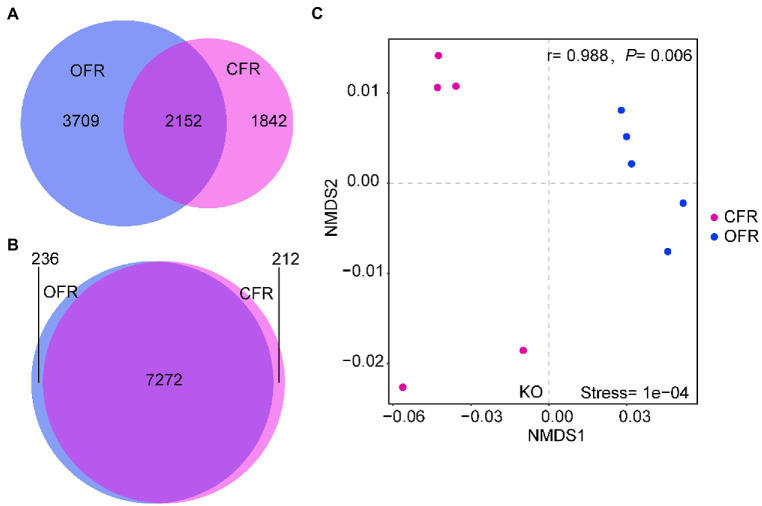
Shifts in rhizosphere functional traits in response to different fertilization management practices. **(A)** Overlap of operational taxonomic units (OTUs) in rhizosphere soil; **(B)** Overlap of functional KOs in rhizosphere soil; and **(C)** Nonmetric multidimensional scaling (NMDS) ordinations of functional genes based on Bray–Curtis distance matrices of KOs.

**Figure 5:**
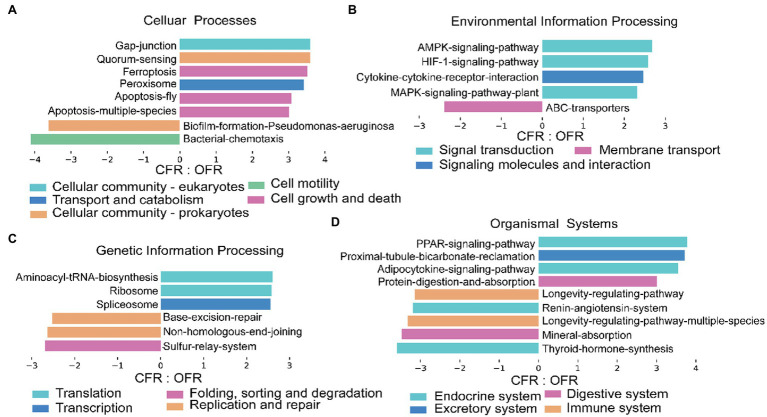
Histograms showing the gene pathways with significant differences between CFR and OFR. **(A-D)** represent functions corresponding to cellular processes, environmental processes, general processes and organic systems, respectively. There is a description above each figure, and it would be redundant to describe it in the title. Other similar studies also operate in this way (Schmidt et al., 2019).

According to the 16S RNA gene sequencing results, there was a clear differentiation among the composition of the rhizosphere microbial community under the different fertilizer treatments. To assess the impact of microbial community changes on rhizosphere function (*via* KEGG), PICRUSt was used to predict the function of the 16S rRNA gene sequence. The tomato plant rhizosphere bacterial community that developed in response to the different fertilizer treatments were more similar in terms of function than taxonomic classification (Figures 4A,B). The PICRUSt analysis indicated that the functional composition of the CFR microbiome differed significantly from that of the OFR (ANOSIM: *r* = 0.988, *p* = 0.006, Figure 4C). In addition, the different abundances of microbiome functional traits were subjected to LEfSe difference analysis. Functions corresponding to cellular processes, including gap junction, quorum, ferroptosis, apoptosis, and peroxisome, showed the greatest difference between the different fertilization management treatments (Figure 5). However, of the functions corresponding to lipid metabolism, only the synthesis and degradation of ketone bodies was enriched in the CFR (results not shown). The particular functions enriched in the OFR include gap junction, quorum sensing, ferroptosis, the AMPK signaling pathway, aminoacyl tRNA biosynthesis, the PPAR signaling pathway and so forth. The particular functions enriched in the CFR mainly include biofilm, chemotaxis, ABC transporters, sulfur relay system, mineral absorption and so forth (Figure 5).

## Discussion

The application of OFs to reduce the incidence of various soil-borne pathogens has been widely reported (Zaccardelli et al., 2010; Avilés and Borrero, 2017; Chilosi et al., 2017). In contrast, the effects of OF application on plant diseases caused by foliar pathogens have rarely been studied. Ntougias et al. (2008) found that the application of OF can induce systemic resistance and significantly reduce the incidence of foliar diseases. However, these studies did not describe the effects of OF application on soil or rhizosphere microbial communities, nor did they describe the potential disease-suppressing microbiota and the potential mechanisms of disease suppression.

In this study, we analyzed the effects of continuous application of OF on the incidence of tomato foliar disease. Compared with continuous application of *CF*, continuous application of OF significantly reduced the incidence of tomato foliar diseases (Figure 1). These results are consistent with the findings of Liu et al. (2018). For bacteria, higher bacterial richness and diversity were detected in the bulk and rhizosphere soils amended with OF compared with *CF* (Figure 2A). For fungi, the bulk and rhizosphere soil diversity were significantly higher in the OF treatment than in the *CF* treatment, but there was no significant difference between richness (Figure 2A), which was consistent with the findings of Xiong et al. (2017a). Previous studies have shown that organic amendments can increase bulk soil and rhizosphere soil bacterial diversity, but no a significant correlation between bacterial diversity and disease incidence has been reported (Ling et al., 2014; Fu et al., 2017). Increased soil microbial diversity plays a key role in promoting soil health and quality (Chaparro et al., 2012; Chourasiya et al., 2017). In this study, we attempted to determine the association between microbial richness or diversity and disease incidence, and the linear models showed a significant negative correlation between disease incidence and bacterial richness and diversity (Supplementary Figure S1). These results suggested that disease suppression is closely related to increased bacterial diversity and richness (de Boer et al., 2003; Garbeva et al., 2004). In addition, fungal richness and diversity were also negatively correlated with disease incidence, but the correlation was not significant (Supplementary Figure S1). Previous studies have shown that application of OF is beneficial for the development of general disease suppression traits because highly diverse communities compete for nutrients with plant pathogens (van Elsas et al., 2012). Moreover, plants infected with foliar diseases can also recruit certain protective rhizosphere microbiota that induce systemic resistance against pathogenic bacteria (Berendsen et al., 2018; Yuan et al., 2018). Therefore, the increase in bacterial richness and diversity after continuous application of OF may contribute to an increased abundance of certain protective rhizosphere microbiota, and together, they induce systemic resistance against plant foliar pathogens.

The PCoA results showed that the structures of the bacterial and fungal communities in the bulk soil and rhizosphere soil of the *CF* and OF treatments were greatly different along the first principle component, and the similarity analysis indicated that the difference was statistically significant, which was similar to the results of a previous study showing that different fertilization management often results in significant differences in soil microbial community structure (Marschner et al., 2003; Zhang et al., 2012). Interestingly, there were also significant differences in the structures of the bacterial and fungal communities in the bulk soil and rhizosphere soil between the two fertilizer treatments along the second principal component (Figure 2B). These results revealed a general rhizosphere effect of the plant (Kowalchuk et al., 2002; Yuan et al., 2018). In conclusion, continuous application of OF appears to serve as a major determining factor of soil microbial community structure, which may further influence soil microbial interactions, possibly with a potential promoting effect to help reduce plant foliar diseases.

Proteobacteria, Actinobacteriota, Bacteroidota, Firmicutes, Acidobacteriota, and Verrucomicrobiota were the dominant bacterial phyla in all the treatment groups, which is general similar to the results of previous studies (Mendes et al., 2011; Shen et al., 2015). For fungi, only Ascomycota was the major fungal phylum (Supplementary Figure S2). Microbiome-mediated disease suppression is often attributed to changes in the presence or abundance of particular microbial populations (Kinkel et al., 2011; Bonilla et al., 2012). We observed that, compared with the application of *CF*, the application of OF significantly increased the abundance of Acidobacteriota and Verrucomicrobiota (Supplementary Figure S2). Xiong et al. (2017b) found that Acidobacteriota and Verrucomicrobiota were relatively more abundant in suppressive soil than in conducive soil. These results suggested that OF application increased the abundance of beneficial microorganisms in the soil. In this study, Mantel tests revealed that the bacterial (*r* = 0.274, *p* < 0.05) and fungal (*r* = 0.296, *p* < 0.05) compositions were significantly correlated with disease incidence, which is in agreement with the results of a previous study (Fu et al., 2017). The results also indicated that the application of OF to reshape the soil microbial community structure indirectly inhibited the incidence of plant foliar diseases.

Spearman rank correlation analysis revealed several specific microbial taxa that were significantly negatively correlated with disease incidence (Supplementary Table S2), such as *Luteolibacter*, *Glycomyces*, *Flavobacterium*, and *Flavihumibacter*, which were significantly more abundant in the OFR than in the CFR (Supplementary Table S2; Figure 3A). Microbial molecular ecological network analysis also showed that *Luteolibacter* and *Glycomyces*, which are negatively correlated with disease incidence, are important hub bacteria in the OFR network (Supplementary Tables S2, S4). *Luteolibacter* and *Glycomyces* belong to *Verrucomicrobiota* and *Actinobacteriota*, respectively, and no specific biocontrol effects of these bacteria against plant pathogens have been reported. However, previous reports have shown that the phyla *Verrucomicrobiota* and *Actinobacteriota* can be used as predictive marker microorganisms of disease-suppressive soils (Trivedi et al., 2017; Xiong et al., 2017b). Therefore, *Luteolibacter* and *Glycomyces* abundance was negatively correlated with tomato foliar disease incidence, suggesting that these taxa may be beneficial new ones for plant protection. Previous studies have confirmed that ISR triggered by *Pseudomonas simiae* WCS 417 leads to the recruitment of *Flavobacterium*, which can induce plant resistance (Sommer et al., 2021). Moreover, *Flavobacterium* can produce indole compounds, biosurfactants and 2,4-di-tert-butylphenol, which may provide potent biocontrol activity against pathogens (Sang and Kim, 2012). *Flavihumibacter* belongs to Bacteroidota and has the potential to inhibit *Ralstonia solanacearum* (Deng et al., 2021). Overall, there was a significant negative correlation between *Luteolibacter*, *Glycomyces*, *Flavobacterium*, and *Flavihumibacter* and disease incidence, indicating that these microbes may be the key taxa for improving the ability of plants to resist pathogenic bacteria. In addition, these genera were abundant in the OFR; therefore, we speculate that OF application may be an effective way to improve plant disease resistance. The different fertilizer management practices significantly altered the soil physicochemical properties (Table 1) and compared with *CF*, OF significantly improved the soil EC, OM content, and AK content. In this study, the Mantel test showed that changes in physicochemical properties had no significant effect on the disease incidence. However, Mantel tests showed that soil physiochemical properties were significantly associated with bacterial and fungal community composition (*r* = 0.45, *p* < 0.001 for bacteria and *r* = 0.64, *p* < 0.001 for fungi). Previous studies have reported that pH, EC, and OM content are generally negatively correlated with soil-borne disease severity (Yadessa et al., 2010; Orr and Nelson, 2018), which was inconsistent with our results. This may be because OF application alters soil physicochemical properties and then alters the composition of the soil microbial community, which indirectly induces plant resistance. Spearman rank correlation test was used to further analyze the correlations between sensitive genera and soil physicochemical properties. The majority of sensitive genera were significantly correlated with pH, OM, AK content, EC, and NH_4_^+^-N content (Supplementary Figure S3). This may explain why the application of OFs increases plant resistance. Finally, the functional prediction results (based on DNA) showed that the application of OF and *CF* had a significant effect on the microbial function within the plant rhizosphere (Figure 5). The results showed that high microbiome diversity in the OFR could ensure improved involvement in multiple ecosystem functions (Gao et al., 2021).

## Conclusion

In summary, the results of this study demonstrated *via* a 2-cycle experiment that, compared with that of *CF*, continuous application of OF progressively and significantly decreased tomato foliar disease. Different fertilizer regimes significantly affected the bulk and rhizosphere microbial community composition. Compared with *CF* application, OF application increased the relatively abundance of more beneficial microbial taxa in the plant rhizosphere, which may provide better protection for the host plants.

## Data Availability Statement

The datasets presented in this study can be found in online repositories. The names of the repository/repositories and accession number(s) can be found at: https://www.ncbi.nlm.nih.gov/, PRJNA830599.

## Author Contributions

XL and YY conceived and designed the study. FH conducted experiments and wrote the manuscript. LL, CM, and JS analyzed the data. All authors contributed to the article and approved the submitted version.

## Funding

This work was supported by the Modern Agro-Industry Technology Research System (CARS-40) and the Science and Technology Program of Guangdong province, China (2020B1212060060).

## Conflict of Interest

The authors declare that the research was conducted in the absence of any commercial or financial relationships that could be construed as a potential conflict of interest.

## Publisher’s Note

All claims expressed in this article are solely those of the authors and do not necessarily represent those of their affiliated organizations, or those of the publisher, the editors and the reviewers. Any product that may be evaluated in this article, or claim that may be made by its manufacturer, is not guaranteed or endorsed by the publisher.

## Footnotes

^1^ http://ccb.jhu.edu/software/FLASH/

^2^ https://www.arbsilva.de/for16S/18S

^3^ https://unite.ut.ee/

## Supplementary Material

The Supplementary Material for this article can be found online at: https://www.frontiersin.org/articles/10.3389/fmicb. 2022.939911/full#supplementary-material

Supplementary Figure S1 Spearman rank correlations between bacterial richness (Chao 1), bacterial diversity (Shannon), fungal richness (Chao 1), and fungal diversity (Shannon) and tomato disease incidence.

Supplementary Figure S2 Bacterial **(A)** and fungal **(B)** community composition at the phylum level. CFB, chemical fertilizer-amended bulk soil; CFR, chemical fertilizer-amended rhizosphere soil; OFB, organic fertilizer-amended bulk soil; and OFR, organic fertilizer-amended rhizosphere soil.

Supplementary Figure S3 Spearman rank correlations between sensitive bacterial genera and physicochemical properties.
