# Supplementary figures and images for: Organic Fertilizer Application Mediates Tomato Defense Against Pseudomonas syringae pv. Tomato, Possibly by Reshaping the Soil Microbiome
Source: Front Microbiol. 2022 Jun 21;13:939911. doi: 10.3389/fmicb.2022.939911 (PMC9253564; doi:10.3389/fmicb.2022.939911)

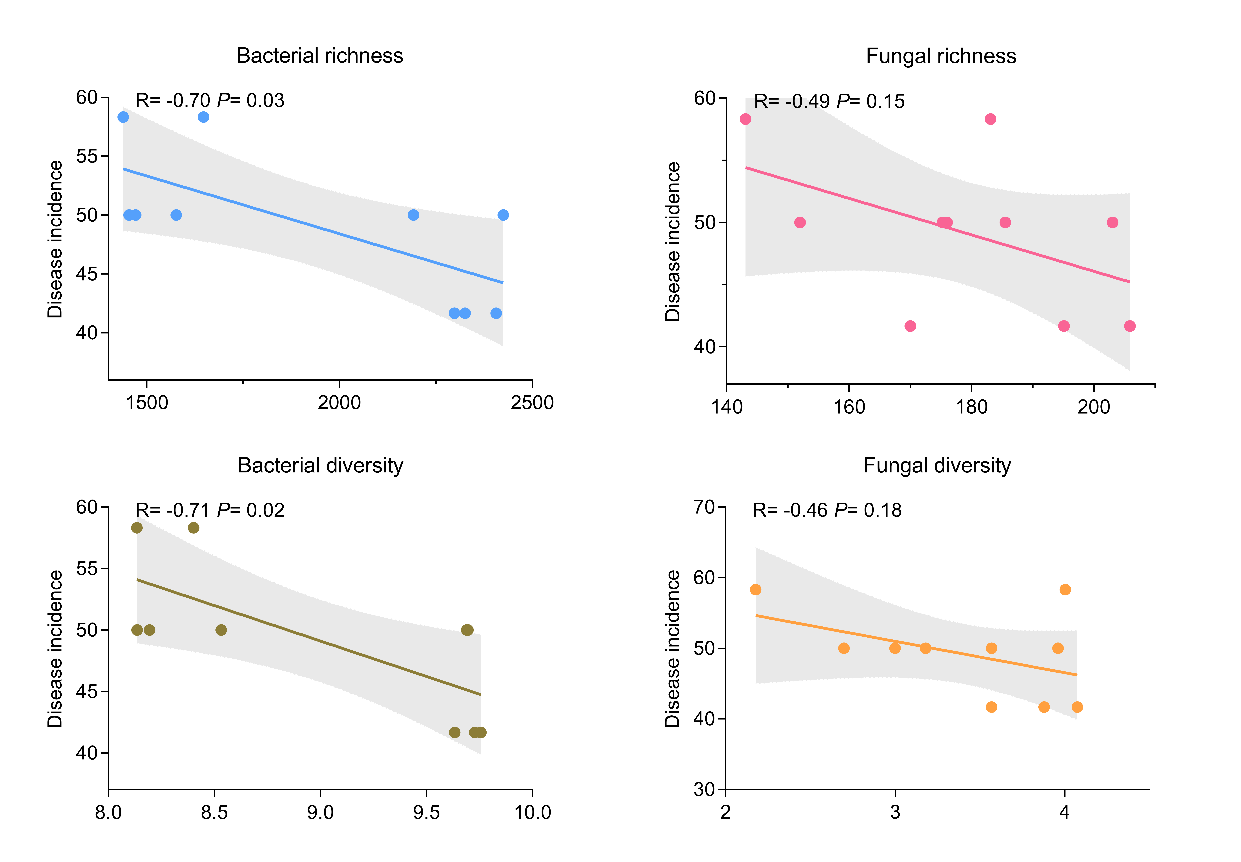

Supplement: Supplementary Figure S1 — Spearman rank correlations between bacterial richness (Chao 1), bacterial diversity (Shannon), fungal richness (Chao 1), and fungal diversity (Shannon) and tomato disease incidence. [file Image_1.TIF]

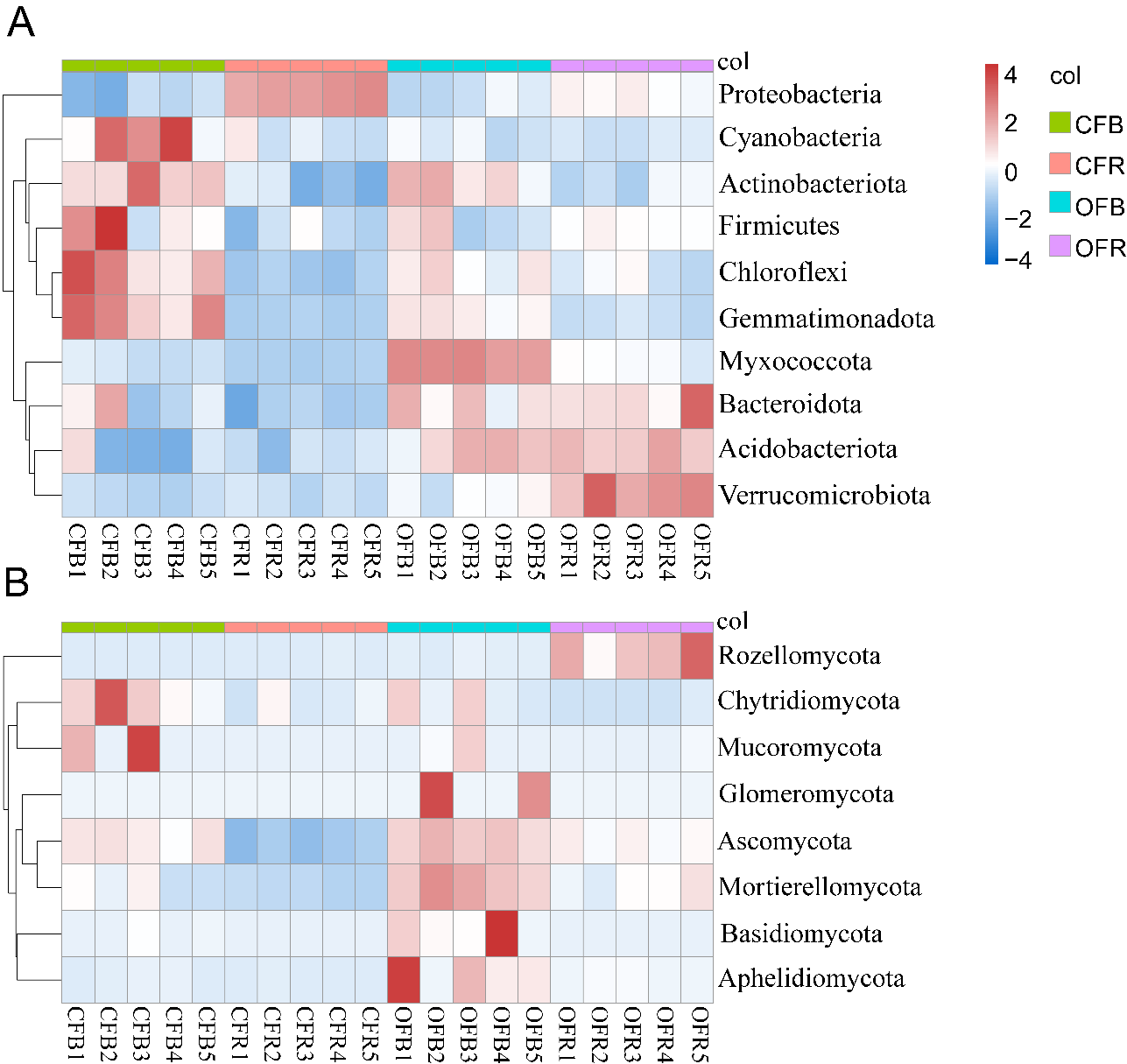

Supplement: Supplementary Figure S2 — Bacterial (A) and fungal (B) community composition at the phylum level. CFB, chemical fertilizer-amended bulk soil; CFR, chemical fertilizer-amended rhizosphere soil; OFB, organic fertilizer-amended bulk soil; and OFR, organic fertilizer-amended rhizosphere soil. [file Image_2.TIF]

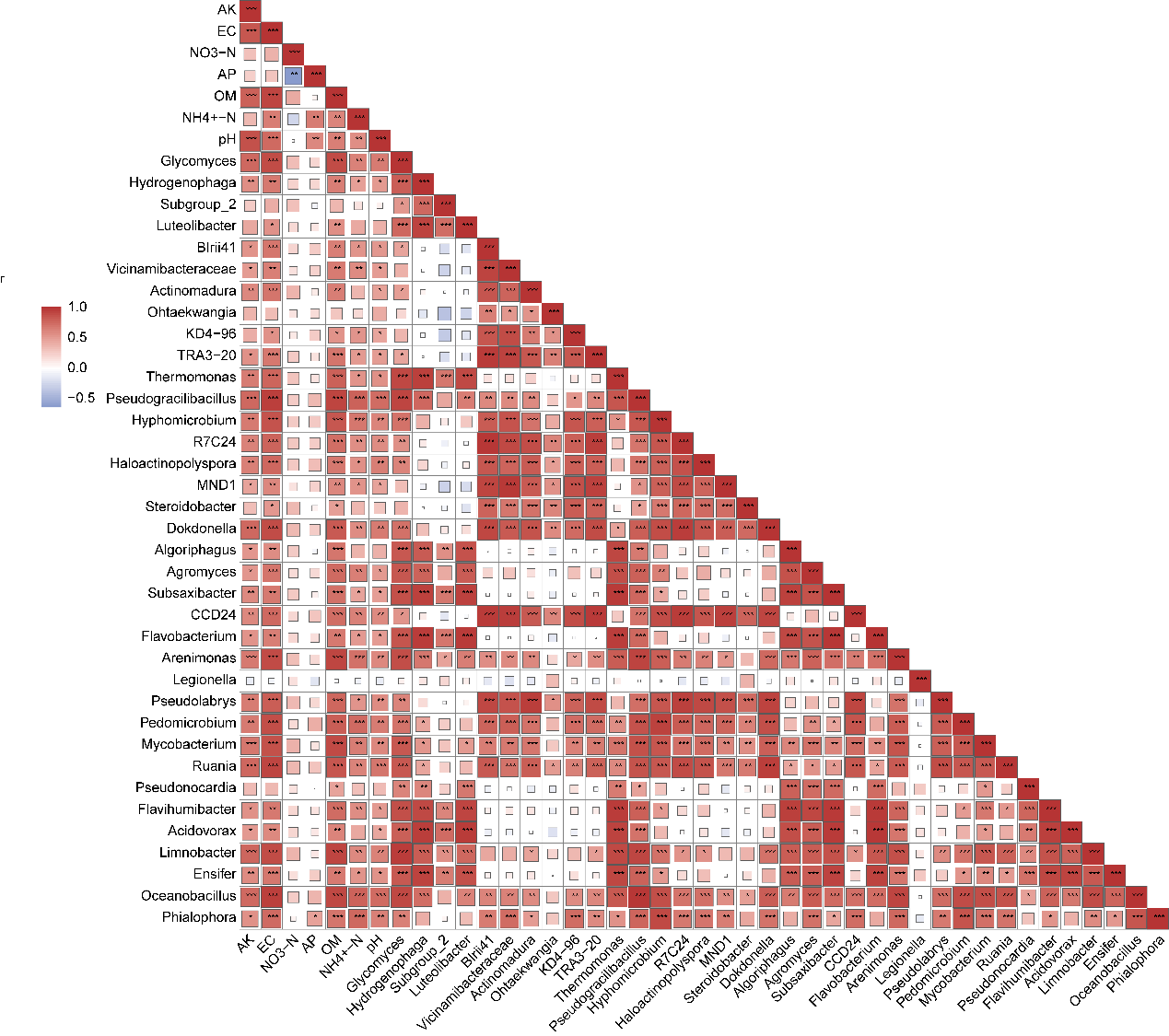

Supplement: Supplementary Figure S3 — Spearman rank correlations between sensitive bacterial genera and physicochemical properties. [file Image_3.TIF]
