# Supplementary material for: Organic Fertilizer Application Mediates Tomato Defense Against Pseudomonas syringae pv. Tomato, Possibly by Reshaping the Soil Microbiome
Source: Front Microbiol. 2022 Jun 21;13:939911. doi: 10.3389/fmicb.2022.939911 (PMC9253564; doi:10.3389/fmicb.2022.939911)
Supplement: Supplementary file 4 [file Data_Sheet_1.docx]

*Supplementary Tables S1-S4*

**Organic fertilizer application mediates tomato defense against foliar pathogens, possibly by reshaping the soil microbiome**

Feng Huang ^1^, Chunhao Mo ^1^, Linfei Li ^1^, Jingling Shi ^1^, Yiwen Yang ^1^, Xindi Liao ^1,2,3*^

^1^ College of Animal Science, South China Agricultural University, Guangzhou 510642, China

^2^ Guangdong Provincial Key Lab of Agro-Animal Genomics and Molecular Breeding, and Key Laboratory of Chicken Genetics, Breeding and Reproduction, Ministry Agriculture, Guangzhou 510642, Guangdong, China

^3^ National-Local Joint Engineering Research Center for Livestock Breeding, Guangzhou 510642, Guangdong, China

* Corresponding authors:

Prof. Xindi Liao, Fax: +86-133-92618303; E-mail: [xdliao@scau.edu.cn](mailto:xdliao@scau.edu.cn)

**Table S1** The disease incidence of bacterial speck disease in tomato in the CF and OF treatments.

| Cycle number | Disease incidence in each treatment (± SD) | | *P* |
| --- | --- | --- | --- |
|  | CF | OF |  |
| Cyele 1 | 71.67±4.08 | 68.33±6.41 | 0.48 |
| Cycle 2 | 53.33±4.10 | 45.00±4.08 | 0.03 |

Note: CF: chemical fertilizer; OF: organic fertilizer; SD: standard deviation. **P* < 0.05; ***P* < 0.01 and ****P* < 0.001.

**Table S2** Spearman rank correlations between sensitive bacterial genera and disease incidence

|  | Phylum | Genus | Relative abundance % | | Sperman correlation | |
| --- | --- | --- | --- | --- | --- | --- |
|  |  |  | CFR | OFR | r | *P* |
| Bacteria | *Proteobacteria* | *Hydrogenophaga* | 0.35 | 3.81 | -0.693 | 0.026 |
|  | *Verrucomicrobiota* | *Luteolibacter* | 0.43 | 2.84 | -0.754 | 0.012 |
|  | *Actinobacteriota* | *Glycomyces* | 0.28 | 2.74 | -0.708 | 0.022 |
|  | *Bacteroidota* | *Subsaxibacter* | 0.02 | 0.97 | -0.717 | 0.019 |
|  | *Bacteroidota* | *Algoriphagus* | 0.04 | 0.95 | -0.646 | 0.044 |
|  | *Proteobacteria* | *Thermomonas* | 0.15 | 0.90 | -0.715 | 0.020 |
|  | *Bacteroidota* | *Flavobacterium* | 0.06 | 0.84 | -0.750 | 0.012 |
|  | *Firmicutes* | *Pseudogracilibacillus* | 0.06 | 0.74 | -0.761 | 0.011 |
|  | *Myxococcota* | *BIrii41* | 0.09 | 0.52 | -0.690 | 0.027 |
|  | *Proteobacteria* | *Acidovorax* | 0.06 | 0.49 | -0.736 | 0.015 |
|  | *Bacteroidota* | *Flavihumibacter* | 0.04 | 0.49 | -0.756 | 0.011 |
|  | *Actinobacteriota* | *Agromyces* | 0.22 | 0.46 | -0.668 | 0.035 |
|  | *Proteobacteria* | *Hyphomicrobium* | 0.12 | 0.45 | -0.697 | 0.025 |
|  | *Acidobacteriota* | *Vicinamibacteraceae* | 0.11 | 0.42 | -0.740 | 0.014 |
|  | *Proteobacteria* | *Arenimonas* | 0.17 | 0.42 | -0.801 | 0.005 |
|  | *Actinobacteriota* | *Actinomadura* | 0.10 | 0.40 | -0.731 | 0.016 |
|  | *Proteobacteria* | *Dokdonella* | 0.07 | 0.39 | -0.661 | 0.037 |
|  | *Actinobacteriota* | *Haloactinopolyspora* | 0.07 | 0.37 | -0.719 | 0.019 |
|  | *Chloroflexi* | *KD4-96* | 0.10 | 0.34 | -0.686 | 0.028 |
|  | *Proteobacteria* | *R7C24* | 0.05 | 0.31 | -0.717 | 0.019 |
|  | *Proteobacteria* | *Limnobacter* | 0.05 | 0.31 | -0.704 | 0.023 |
|  | *Actinobacteriota* | *Ruania* | 0.03 | 0.30 | 0.712 | 0.021 |
|  | *Proteobacteria* | *TRA3-20* | 0.08 | 0.29 | -0.639 | 0.047 |
|  | *Proteobacteria* | *Ensifer* | 0.05 | 0.29 | -0.753 | 0.012 |
|  | *Actinobacteriota* | *Mycobacterium* | 0.11 | 0.29 | -0.707 | 0.022 |
|  | *Proteobacteria* | *CCD24* | 0.07 | 0.29 | -0.699 | 0.024 |
|  | *Proteobacteria* | *Pedomicrobium* | 0.10 | 0.28 | -0.747 | 0.013 |
|  | *Firmicutes* | *Oceanobacillus* | 0.02 | 0.27 | -0.733 | 0.016 |
|  | *Actinobacteriota* | *Pseudonocardia* | 0.12 | 0.27 | -0.668 | 0.034 |
|  | *Proteobacteria* | *Legionella* | 0.15 | 0.26 | -0.651 | 0.041 |
|  | *Bacteroidota* | *Ohtaekwangia* | 0.17 | 0.26 | -0.724 | 0.018 |
|  | *Proteobacteria* | *Pseudolabrys* | 0.06 | 0.23 | -0.740 | 0.014 |
|  | *Proteobacteria* | *MND1* | 0.11 | 0.22 | -0.696 | 0.025 |
|  | *Acidobacteriota* | *Subgroup_2* | 1.17 | 2.34 | -0.639 | 0.046 |
|  | *Proteobacteria* | *Steroidobacter* | 0.10 | 0.27 | -0.748 | 0.012 |
| Fung | *Ascomycota* | *Phialophora* | 0.02 | 0.48 | -0.7172 | 0.0195 |

**Table S3** Topological properties of co-occurring bacterial networks in the rhizosphere of the CFR and OFR

| Network Indexes | Treatment | |
| --- | --- | --- |
|  | CFR | OFR |
| Total nodes | 95 | 99 |
| Total links | 545 | 517 |
| Number of positive correlations | 360 | 310 |
| Number of negative correlations | 185 | 207 |
| Average clustering coefficient | 0.541 | 0.526 |
| Connect components | 4 | 3 |
| Modularity | 0.661 | 2.256 |
| Average degree | 4.356 | 10.44 |
| Average path length | 6.922 | 3.493 |

**Table S4** Taxonomic positions of the top 10 hubs in CFR and OFR bacterial networks

| Bacteria |  | Taxonomic position |
| --- | --- | --- |
| Top hubs in CFR | ASV21 | Bacteria--Bacteroidota--Bacteroidia--Cytophagales--Hymenobacteraceae--Pontibacter |
|  | ASV38 | Bacteria--Actinobacteriota--Actinobacteria--Streptomycetales--Streptomycetaceae--Streptomyces |
|  | ASV223 | Bacteria--Proteobacteria--Gammaproteobacteria--Xanthomonadales--Xanthomonadaceae--Lysobacter |
|  | ASV31 | Bacteria--Proteobacteria--Alphaproteobacteria--Rhizobiales--Rhizobiaceae |
|  | ASV106 | Bacteria--Proteobacteria--Alphaproteobacteria--Sphingomonadales--Sphingomonadaceae--Sphingomonas |
|  | ASV160 | Bacteria--Proteobacteria--Gammaproteobacteria--Pseudomonadales--Pseudomonadaceae--Pseudomonas |
|  | ASV122 | Bacteria--Actinobacteriota--Actinobacteria--Micrococcales--Cellulomonadaceae--Actinotalea |
|  | ASV177 | Bacteria--Proteobacteria--Alphaproteobacteria--Rhizobiales--Rhizobiaceae--Allorhizobium |
|  | ASV235 | Bacteria--Firmicutes--Bacilli--Alicyclobacillales--Alicyclobacillaceae--Tumebacillus |
|  | ASV44 | Bacteria--Actinobacteriota--Actinobacteria--Micrococcales--Intrasporangiaceae |
| Top hubs in OFR | ASV123 | Bacteria--Actinobacteriota--Actinobacteria--Propionibacteriales--Nocardioidaceae--Nocardioides |
|  | ASV30 | Bacteria--Proteobacteria--Alphaproteobacteria--Rhizobiales--Rhizobiaceae |
|  | ASV188 | Bacteria--Proteobacteria--Alphaproteobacteria--Sphingomonadales--Sphingomonadaceae |
|  | ASV10 | Bacteria--Proteobacteria--Gammaproteobacteria--Xanthomonadales--Xanthomonadaceae--Lysobacter--Lysobacter erysipheiresistens |
|  | ASV12 | Bacteria--Proteobacteria--Gammaproteobacteria--Burkholderiales--Oxalobacteraceae--Noviherbaspirillum |
|  | ASV4 | Bacteria--Actinobacteriota--Actinobacteria--Glycomycetales--Glycomycetaceae--Glycomyces |
|  | ASV88 | Bacteria--Proteobacteria--Alphaproteobacteria--Sphingomonadales--Sphingomonadaceae--Sphingobium |
|  | ASV7 | Bacteria--Proteobacteria--Alphaproteobacteria--Sphingomonadales--Sphingomonadaceae |
|  | ASV333 | Bacteria--Verrucomicrobiota--Verrucomicrobiae--Verrucomicrobiales--Rubritaleaceae--Luteolibacter |
|  | ASV86 | Bacteria--Proteobacteria--Alphaproteobacteria--Rhizobiales--Devosiaceae--Devosia |
